# Pittsburgh Sleep Quality Index (PSQI) Changes in Virologically Suppressed People Living with HIVSwitching to Long-Acting Cabotegravir and Rilpivirine

**DOI:** 10.3390/biomedicines12091995

**Published:** 2024-09-02

**Authors:** Nicolò De Gennaro, Mariacristina Poliseno, Angelo Dargenio, Flavia Balena, Deborah Fiordelisi, Vito Spada, Greta Romita, Giacomo Guido, Francesco Di Gennaro, Giuseppe Bruno, Mariantonietta Purgatorio, Giovanni Battista Buccoliero, Annalisa Saracino

**Affiliations:** 1 Clinic of Infectious Diseases, Department of Precision and Regenerative Medicine and Ionian Area (DiMePRe-J), University of Bari “Aldo Moro”, 70124 Bari, Italy; nico84degennaro@alice.it (N.D.G.); angelo.dargenio94@gmail.com (A.D.); flaviabalena89@gmail.com (F.B.); deborah.fiordelisi88@gmail.com (D.F.); v.spada@studenti.uniba.it (V.S.); greta.romita@gmail.com (G.R.); giacguido@gmail.com (G.G.); francesco.digennaro1@uniba.it (F.D.G.); annalisa.saracino@uniba.it (A.S.); 2 Infectious Diseases Unit, San Giuseppe Moscati Hospital, Azienda Sanitaria Locale Taranto, 74121 Taranto, Italy; giusbruno85@gmail.com (G.B.); mariantoniettapurgatorio@yahoo.it (M.P.); giovannibattis.buccoliero@asl.taranto.it (G.B.B.)

**Keywords:** cabotegravir, sleep quality, PSQI, antiretroviral treatment, HIV

## Abstract

Background: Limited evidence is available about sleep quality changes associated with the use of Cabotegravir (CAB), a new, long-acting (LA) antiretroviral (ARV) drug belonging to the class of Integrase Strand Transfer Inhibitors (INSTIs). Methods: Pittsburgh Sleep Quality Index (PSQI) was calculated in 53 people living with HIV (PLWH) under the care of the outpatient services of two Italian Infectious Diseases Centers in Apuliabefore (M0) and seven months after (M7) the switch to LA CAB. Global scores and relative subitems were compared using paired sample tests. The same analysis was repeated in subgroups of PLWH switching from INSTIs-, Dolutegravir-(DTG), and Bictegravir (BIC)-based regimens. Results: A significant reduction was reported in global mean (±StandardDeviation, SD) PSQI at M7 compared to M0 (4 (±3) vs. 3 (±2), *p* = 0.01), particularly in the areas of sleep latency and sleep disturbances. The improvement was also significant in PLWH already on INSTIs- (from median 3 to 2 points, *p* = 0.02) and DTG-based (from median 4 to 2, *p* = 0.01) ARV regimens, but not among those who switched from BIC-based regimens. Conclusions: PLWH reported improved sleep quality after switching from ARV treatment to LA CAB. Further studies are needed to give deeper insights into this phenomenon.

## 1. Introduction

Human Immunodeficiency Virus (HIV) infection currently affects approximately 40 million people globally, with about 130,000 individuals living with the virus in Italy alone [1]. Despite a steady rate of new diagnoses in recent years, significant improvements have been observed in both life expectancy and quality of life for people living with HIV (PLWH). These advancements over the past two decades can be largely attributed to the development and widespread use of modern antiretroviral therapy (ART), which combine high efficacy with manageable side effects [2,3].

Among these therapies, Integrase Strand Transfer Inhibitors (INSTIs) represent a particularly effective class of antiretroviral medications. INSTIs are renowned for their robust efficacy in suppressing HIV viral replication and for their generally favorable safety profiles [4]. Second-generation INSTIs, such as Dolutegravir (DTG) and Bictegravir (BIC), have emerged as preferred options for the initiation and modification of ART. These agents are favored in middle- and high-income countries due to their potent antiviral activity and low incidence of adverse effects [2,5]. Recent guidelines and clinical studies underscore the role of DTG and BIC in optimizing HIV treatment regimens and improving patient outcomes [6,7].

Despite their general tolerability, INSTIs have been associated with minor adverse effects [8], including weight gain, alterations in lipid profiles, and Central Nervous System (CNS) disorders. Recent data indicate a potential relation between INSTIs and sleep disturbances, such as insomnia and nightmares, particularly with second-generation INSTIs [9]. These adverse events are typically of mild to moderate severity, with DTG being the most frequently implicated ARV drug, especially among women, and the elderly, and when co-formulated with Abacavir (ABC) [10].

Cabotegravir (CAB), an injectable, long-acting (LA) INSTIs, has recently become available in co-formulation with Rilpivirine (RPV) for the treatment of ART-experienced, virologically suppressed PLWH. 

Although clinical trials have demonstrated a good safety and tolerability profile of LA CAB + RPV [11,12,13], there is a lack of information regarding the effects that this medication, characterized by a unique molecular structure and route of administration, might have on the CNS, and particularly on sleep quality. 

In light of this, our study aimed to investigate the effect on sleep quality among a cohort of ART-experienced PLWH who switched from their oral ART regimen to injectable LA CAB + RPV, using a validated survey tool.

## 2. Materials and Methods

This prospective observational study was conducted at the HIV outpatient clinics of the University Hospital Policlinico of Bari (Bari, Italy) and the San Giuseppe Moscati Hospital (Taranto, Italy). From 1 February 2023 to 31 May 2024, all ART-experienced PLWH who were consecutively transitioning from any ART regimen to LA CAB+RPV, based on eligibility criteria, were assessed for their sleep quality using the Pittsburgh Sleep Quality Index (PSQI) questionnaire.

The PSQI is a screening tool that evaluates sleep quality and disturbances over a one-month interval. It comprises seven components: (i) subjective sleep quality; (ii) sleep latency; (iii) sleep duration; (iv) sleep efficiency; (v) sleep disturbances; (vi) use of sleeping medications; and (vii) daytime dysfunction. Each component is scored, and the sum yields a global score ranging from 0 to 21; a score > 5 indicates poor sleep quality [14].

Both the global PSQI score and scores for individual components were assessed before switching to LA CAB + RPV (Month 0, M0) and again at the 5th drug injection, seven months later (Month 7, M7).

A total of 53 patients, who completed seven months of therapy during the study period (considered a sufficiently appropriate duration), were included in the analysis. The sample size was determined based on a 95% confidence level, a 5% margin of error, and an assumed population proportion of 50%, due to the lack of precise prevalence data regarding sleep disorders in HIV-positive individuals. Using these parameters, the minimum required sample size was calculated to be 47 patients.

Participation in the survey was voluntary, and consent was implied through the completion of the questionnaire; therefore, no formal written consent was required. Ethics approval was obtained from the Policlinic of Bari, Italy (approved n.7147 by 1 December 2022). This study was conducted following the Declaration of Helsinki.

Baseline demographic, clinical, biochemical, and immunovirological data of all study participants with available PSQI scores at M0 and M7 by 31 May 2024 were collected. Chi-square tests and Student′s *t*-tests or Mann–Whitney U-tests were used to compare categorical and continuous variables, respectively, to test the null hypothesis of no difference between PLWH with baseline PSQI scores < 5 and ≥5 points.

A Paired Samples Student′s *t*-test/Wilcoxon Rank Sum test was used, as appropriate, to compare PSQI values reported by the study population at the two time points both overall and for each subitem. This analysis was repeated in subgroups of PLWH categorized according to their previous ART regimen (INSTIs-, DTG-, and BIC-based).

Statistical analysis was performed using Jamovi 2.3.2 [15], and statistical significance was set at a *p*-value < 0.05.

## 3. Results

Overall, 53 PLWH, predominantly males (47 patients, 89%), were enrolled in the analysis. Their main baseline characteristics are shown in Table 1.

The median patient age was 43 (range 35–70) years, while the median duration of HIV infection was 10 (range 7–13) years. The switch to the LA strategy was motivated by the reduction inpill burden (44/53 patients, 83%), avoidance of drug–drug interactions (DDIs) (8/53 patients, 15%), and in 1 case (2%) by the patient’s preference. At the time of the switch, the population had been virologically suppressed for a median of 7 (range 5–9) years, and 26 PLWH were already on two-drug regimen (2DR) ART.

Before switching to LA CAB + RPV, 40 PLWH were on an INSTIs based regimen. 

Specifically, 25 of them were on DTG-containing ART such as DTG/Lamivudine (3TC) (19/53) or DTG/RPV (6/53). Other INSTIS-based ART regimens included Tenofovir Alafenamide (TAF/FTC)/BIC (11/53), TAF/FTC/Elvitegravir (EVG)/cobicistat (Cobi) (2/53), and Raltegravir (RAL) plus TAF/FTC or ABC/3TC (2/53). Additionally, 12/53 PLWH (23%) switched from TAF/FTC/RPV and 1 from TAF/FTC/Darunavir (DRV)/Cobi. 

Overall, the mean PSQI before the reported treatment switch was 4 (±3). 

Notably, 18 PLWH (34%) had a baseline score above 5 points. There were no significant differences when comparing PLWH with baseline PSQI scores above and below 5 points, except for a slightly higher prevalence of comorbidities among those with higher baseline PSQI (57% vs. 10%, *p* = 0.03).

**Figure 1:**
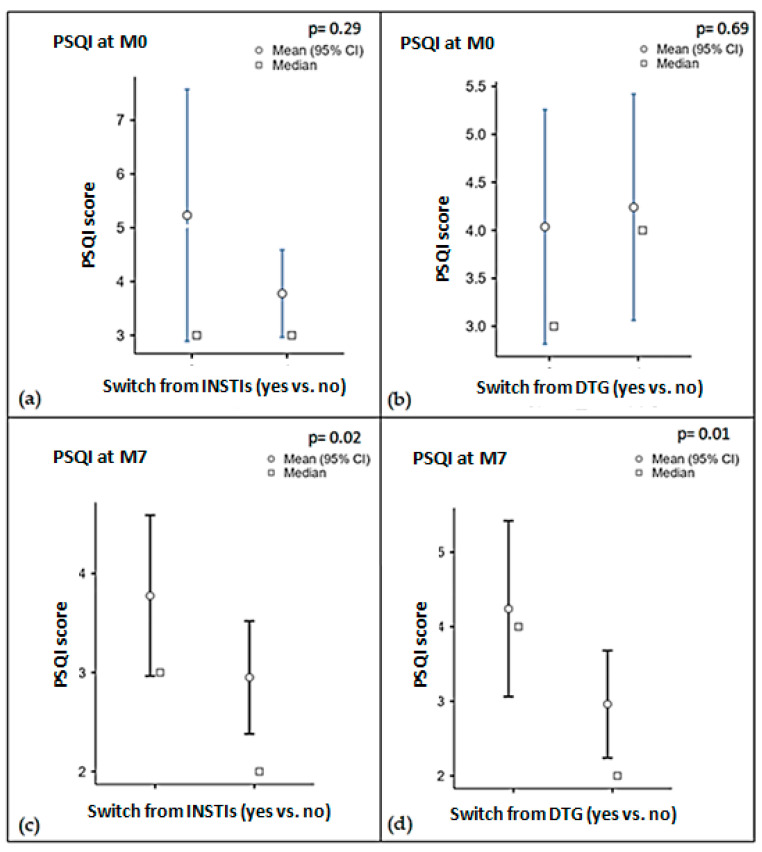
Results of PSQI collected at baseline (M0)) in PLWH switching to CAB + RPV LA from INSTIs- (**a**) and DTG-based ARV regimens (**b**). The analysis was repeated at Month 7 (M7) both in PLWH previously on INSTIs- (**c**) and DTG-based ARV regimens (**d**).

No significant differences in baseline PSQI were observed comparing patients coming from an INSTIs-based versus non-INSTIs-containing ARV regimens (Figure 1a). However, 25 PLWH switching from DTG-based regimens reported higher baseline total PSQI compared to those on other regimens, though this difference was not statistically significant (median 4 vs. 3 points, *p* = 0.69, Figure 1b).

None of the participants reported using sleep medications.

**Figure 2:**
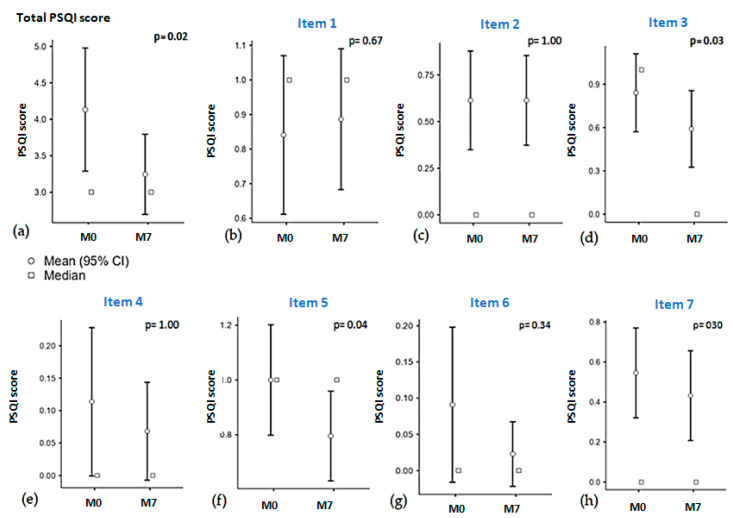
Results of PSQI were collected from 53 PLWH before (M0) and 7 months (M7) after the switch to CAB + RPV LA. Both differences in total score (**a**) and subscores of Item 1 to 7 (**b**–**h**) were outlined.

After 7 months, an overall reduction in PSQI scores was reported (from mean 4 (±3) to 3 (±2) points, *p* = 0.02) (Figure 2a). Observing the scores for each PSQI item, a more significant reduction was noted for Items 3 and 5, regarding sleep duration and sleep disturbances (from mean 0.84 to 0.58 points, *p* = 0.03, and from 1 to 0.83 points, *p* = 0.04, respectively) (Figure 2b–h).

Remarkably, the improvement in PSQI was also evident in subjects switching to LA CAB + RPV from other INSTIs-based regimens (from median 3 to 2 points, *p* = 0.02, Figure 1c), and especially in PLWH who were on DTG (from median 4 to 2 points, *p* = 0.01). This difference was not observed in subjects on TAF/FTC/BIC, for whom instead a slight, non-significant increase in PSQI was reported (from a median of 2 to 3 points, *p* = 0.66).

## 4. Discussion

The introduction of long-acting antiretroviral drugs, specifically Integrase Strand Transfer Inhibitors (INSTIs) with an injectable administration method, has revolutionized the concept of therapy for chronic HIV infection. 

To the best of our knowledge, given the short time since they have been available on the market, this is one of the first studies to assess the impact of long-acting INSTIs on sleep quality. Our data, although limited to our experience, indicate an improvement in sleep quality reported by our patients, including those coming from other INSTIs-based regimens, and particularly those who previously were on a DTG-based regimen.

Data on the efficacy and safety of these drugs in treatment-experienced patients are derived from clinical trials LATTE-1, LATTE-2, ATLAS-2M, and FLAIR [11,12,13]. The most commonly described side effects were injection site reactions nasopharyngitis, upper respiratory tract infection, headache, and diarrhea. They were frequently mild or moderate in severity, leading to treatment discontinuation in a negligible number of participants, as a confirmation of the overall tolerability of the regimen. Notably, no new onset of significant sleep disturbances or other neuropsychiatric disorders was observed in clinical trials.

The prevalence of neuropsychiatric disorders in patients with chronic HIV infection is particularly high [16]. In addition to the genetic and environmental risk factors common to the general population [17,18], there are factors specifically related to HIV infection. Some of these factors are organic in nature, such as irreversible CNS injury caused by HIV before the initiation of ARTs; persistent viral replication in the CNS due to the inability of some ARV drugs to fully penetrate the blood–brain barrier; and ongoing inflammation or toxicity within the CNS as a result of ARTs [19]. Current evidence specifically supports an association between Efavirenz and treatment-emergent CNS adverse events, while emerging data suggest a potential association with the use of INSTIS, and in particular with DTG [9,10,19].

Approximately half of the patients involved in our study were from regimens containing Dolutegravir, and considering those also on Bictegravir and Raltegravir, over 70% of the cohort consisted of individuals taking oral INSTIs. 

A significant discovery is that, despite the relatively young age of the patients, averaging 43 years old, over one-third of the study participants experienced suboptimal sleep quality. Nevertheless, contrary to expectations, baseline data did not show higher PSQI scores among these patients compared to those on non-INSTIs-based regimens. However, our data indicated that switching to LA CAB + RPV had positive effects on patients′ reported sleep quality, especially in those coming from DTG-based ARV regimens.

A contrasting finding was observed in the only study, to our knowledge, published on this topic. In this recent work by Mazzitelli et al. [20], the sleep quality of PLWH, measured using PSQI, deteriorated following the switch to CAB + RPV. However, in the same study, these patients reported higher scores on quality-of-life assessment questionnaires.

This discordant result warrants a more in-depth analysis and prompts reflection on the non-organic factors underlying neuropsychiatric disorders, particularly sleep disturbances. These factors include advanced age, female sex, history of alcohol or drug dependence, and social vulnerability [16]. In addition to these general factors, people living with HIV face unique challenges such as difficulty accepting the diagnosis and chronic treatment, feelings of discrimination related to their condition, and personal and occupational choices influenced by these factors [17,21,22,23]. Such challenges can lead to significant psychological distress, which often manifests as sleep disturbances, as measured by tools like the PSQI. Addressing sleep quality is crucial, as it reflects broader psychological and behavioral adaptations to the chronic stress associated with living with HIV. This approach parallels the use of the Emergency Response and Psychological Adjustment Scale (ERPAS) in evaluating responses to emergencies, underscoring the importance of targeted psychosocial interventions to enhance overall well-being in both HIV patients and individuals facing acute stressors [24].

It has been demonstrated that transitioning to injectable therapies can alleviate the perception of illness in patients with chronic HIV infection [25,26]. In this context, the observed improvement in reported sleep quality among our patients was anticipated and may reflect a broader enhancement in their overall quality of life.

However, the present study is unable to exclude the possibility that biochemical mechanisms, possibly related to the different mode and less frequent administration schedule—characterized by the parenteral route and bi-monthly dosing of the drug—may be responsible for this process. We hope that this study may catalyze future investigations aimed at exploring these mechanisms shortly. Other limitations of this study include the small number of patients and the relatively short follow-up period, which in some cases hindered the observation of statistically significant findings. However, these limitations were largely attributable to the recent introduction of injectable therapy in Italy, which has been available for less than two years. Finally, a limitation of our study could be the fact that sleep quality—an inherently multifactorial condition influenced by various physiological and pathological factors and closely interdependent with psychological well-being—was assessed solely using the PSQI. Evaluating aspects such as anxiety, depression, and neurocognitive disorders through specific surveys could help contextualize the results of this study within a broader framework.

In an era where a multitude of antiretroviral drugs are available, all characterized by comparable efficacy in viral suppression, the clinician’s responsibility lies in addressing issues related to patient aging, comorbidities, drug–drug interactions (DDIs), and pharmacological toxicities [27,28].

In this perspective, this study, utilizing real-world patient data, highlights an additional factor to consider when selecting an innovative regimen for sustaining antiretroviral therapy in ART-experienced patients, such as sleep quality.

## 5. Conclusions

This study highlights the potential benefits of long-acting injectable antiretrovirals, such as CAB + RPV, in improving sleep quality for ART-experienced patients. Despite limitations like a small sample size and a short follow-up, our findings suggest that injectable antiretroviral therapies may enhance overall quality of life, reducing the perception of illness in chronic HIV patients. Clinicians should consider these factors when selecting antiretroviral regimens to ensure comprehensive patient care. Further research is needed to explore the underlying biochemical mechanisms and validate these results in larger cohorts.

## Figures and Tables

**Table 1:** Baseline features of the study population.

Variables	Total (N = 53)	PLWH with Baseline PSQI < 5 (N = 35)	PLWH with Baseline PSQI ≥ 5 (N = 18)	*p*-Value
Male sex, n (%)	47 (89)	15 (83)	32 (91)	0.38
Age, years, mean (SD)	43 (35–70)	40 (35–47)	48 (35–55)	0.18
Italian nationality, n (%)	52 (98)	35 (100)	17 (94)	0.16
Transmission route, n (%) Heterosexual contacts MSM IDU Other	16 (30) 31 (58) 1 (2) 5 (9)	11 (31) 20 (57) 1 (3) 3 (9)	5 (28) 11 (61) 0 (0) 2 (11)	0.87
Non-B HIV-1subtype, n (%)	9 (26)	7 (27)	2 (22)	0.78
HCV-Ab-positive, n (%)	1 (2)	1 (3)	0 (0)	0.51
Comorbidities, n (%) Hypertension Type II Diabetes Dyslipidemia Obesity Depression Osteoporosis	5 (26) 4 (21) 6 (32) 3 (16) 5 (26) 2 (10)	1 (10) 1 810) 4 (40) 1 (10) 2 (20) 1 (10)	4 (44) 3 (33) 2 (22) 2 (22) 3 (33) 1 (11)	0.08 0.21 0.40 0.46 0.51 0.94
≥2 Comorbidities, n (%)	6 (32)	1 (10)	5 (57)	0.03
Body mass index, median (IQR)	24 (23–26)	24 (23–26)	24 (23–28)	0.57
Years of HIV infection, median (IQR)	10 (7–13)	10 (6–12)	9 (7–13)	0.39
Years of ART, median (IQR)	8 (6–10)	7 (6–10)	8 (6–10)	0.62
AIDS diagnosis at baseline, n (%)	5 (94)	2 (6)	3 (17)	0.19
Nadir CD4+, cells/mm^3^, mean (SD)	390 (198)	368 (201)	388 (197)	0.77
Zentih HIV RNA, log_10_copies/mL, median (IQR)	4.2 (3.7–5.0)	4.3 (3.7–5.2)	1.2 (3.7–4.7)	0.98
History of virological failure, n (%)	1 (2)	1 (3)	0 (0)	0.47
Duration of viral suppression, years, median (IQR)	7 (5–9)	6 (5–9)	7 (3–9)	0.88
Switch from 2 DR, n (%)	26 (49)	14 (40)	12 (67)	0.06
Oral lead-in, n (%)	21 (40)	14 (40)	7 (39)	0.93
Reasons for switch, n (%) Pill burden reduction Virological failure with previous ART Toxicity issues with previous ART DDIs Patients′ wish Baseline PSQI, mean (SD)	44 (83) 0 (0) 0 (0) 8 (15) 1 (2) 4 (3)	28 (80) 0 (0) 0 (0) 6 (17) 1 (2) 2 (1)	16 (89) 0 (0) 0 (0) 2 (11) 0 (0) 7 (3)	0.63 <0.001

PSQI: Pittsburg Sleep Quality Index; SD: Standard Deviation; IQR: Inter Quartile Range; HCV: Hepatitis C Virus; MSM: Males who have Sex with Males; IDU: Intravenous Drug Users; ART: antiretroviral therapy; 2DR: 2 drug-regimens; DDIs: drug–drug interactions.

## Data Availability

The original contributions presented in the study are included in the article; further inquiries can be directed to the corresponding author/s.
